# SCOUT: Skull-Corrected Optimization for Ultrasound Transducers

**DOI:** 10.3390/bioengineering11111144

**Published:** 2024-11-13

**Authors:** Zheng Jiang, Michelle Hua, Jacqueline Li, Hieu Le Mau, James Choi, William B. Gormley, Jose M. Amich, Raahil M. Sha

**Affiliations:** 1 Zeta Surgical Inc., Boston, MA 02111, USA; zheng.jiang@zetasurgical.com (Z.J.); michellehua2005@gmail.com (M.H.); jacqueline.li@zetasurgical.com (J.L.); hieu.lemau@zetasurgical.com (H.L.M.); j.choi@imperial.ac.uk (J.C.); jose.amich@zetasurgical.com (J.M.A.); 2 Department of Bioengineering, Imperial College London, London SW7 2AZ, UK; 3 Harvard Medical School, Boston, MA 02115, USA; wgormley@bwh.harvard.edu; 4 Computational Neurosurgical Outcomes Center, Brigham and Women’s Hospital, Boston, MA 02115, USA; 5 Department of Neurological Surgery, Brigham and Women’s Hospital, Boston, MA 02115, USA

**Keywords:** focused ultrasound, transcranial treatment, single-element transducer, transducer placement, optimization algorithm

## Abstract

Transcranial focused ultrasound has been studied for non-invasive and localized treatment of many brain diseases. The biggest challenge for focusing ultrasound onto the brain is the skull, which attenuates ultrasound and changes its propagation direction, leading to pressure drop, focus shift, and defocusing. We presented an optimization algorithm which automatically found the optimal location for placing a single-element focused transducer. At this optimal location, the focus shift was in an acceptable range and the ultrasound was tightly focused. The algorithm simulated the beam profiles of placing the transducer at different locations and compared the results. Locations with a normalized peak-negative pressure (PNP) above threshold were first found. Then, the optimal location was identified as the location with the smallest focal volume. The optimal location found in this study had a normalized PNP of 0.966 and a focal volume of 6.8% smaller than without the skull. A Zeta navigation system was used to automatically place the transducer and track the error caused by movement. These results demonstrated that the algorithm could find the optimal transducer location to avoid large focus shift and defocusing. With the Zeta navigation system, our algorithm can help to make transcranial focused ultrasound treatment safer and more successful.

## 1. Introduction

Transcranial focused ultrasound is being widely studied in therapeutic applications, such as thermal ablation, drug delivery, blood–brain barrier (BBB) disruption, neuro-modulation, and liquid biopsy [1,2,3,4,5]. To achieve therapeutic effects in these applications, ultrasound is focused on a small spot to achieve a high acoustic power or pressure. The high acoustic power or pressure generated then causes a high temperature or mechanical forces (generated by ultrasound along or together with microbubbles) that work on tissues for the treatment. The reasons for generating a high temperature or pressure only at a small spot are for performing a localized treatment and for avoiding overheating/treating healthy tissues surrounding the target and along the propagation pathway.

There are different ways to focus ultrasound. The common approaches are either using multi-element arrays [6,7,8,9] or using single-element focused transducers [10,11,12]. For a multi-element array, its elements can be arranged on a flat surface or on a curved surface. When using a flat array, ultrasound can be focused by changing the phase or time delay of the excitation signals for the elements to ensure the ultrasound generated by the elements arrives at the focal position in phase or at the same time. In transcranial focused ultrasound applications, multi-element arrays are normally arranged in a dome shape (hemispherical shape). This arrangement gives the elements a natural focal point, which is the geometric center of the array. The elements can still be excited with a phase difference or a time delay to steer the focus to a different position or to compensate for the distortion caused by the propagation medium. As for a single-element focused ultrasound transducer, the transducer itself is curved and has a natural focus at the geometric center. When emitting ultrasound, it is automatically focused at a fixed position.

The most significant problem with focusing ultrasound transcranially is the heterogeneity of the propagation medium, which is mainly caused by the presence of the skull. The skull is a layered structure with complex micro structures, making the propagation pathway for ultrasound very different at different locations [13,14,15,16]. The presence of the skull not only attenuates the ultrasound but also distorts the propagation direction of the ultrasound. This leads to a decreased acoustic pressure at the focus, defocusing (a larger focal size), and focus shift. For multi-element arrays, the above-mentioned aberration can be corrected. The defocusing and focus shift problems can be solved by applying a phase difference or time delay to the excitation signals of individual elements [6,9,17]. The acoustic pressure drop at the focus point can be compensated with a higher excitation voltage. However, multi-element arrays are not currently widely used in studies due to their high cost. Single-element focused transducers are still commonly used in various applications for which the aberration caused by the skull is difficult to deal with. In most studies, the aberration caused by the skull is ignored, which can lead to unsuccessful or unsafe treatment. Park et al. attempted to optimize the position of the single-element focused transducer for transcranial applications [18]; however, their study examined only the acoustic pressure drop after penetrating through the skull (which can be compensated for with a higher excitation voltage), while the defocusing and focus shift problems, which play a more important role in ensuring successful and safe treatment, are not addressed. The same group presented another method that takes the focal shit into account, but this method requires simulating tens of thousands of locations, which is time-consuming hence not very practical in real practice [19]. This method would require much more time when dealing with the high frequencies used in real treatments. Choi et al. presented a deep neural network for placing a single-element transducer [20]. This method provides real-time visualization of the ultrasound focus; hence, a good location for placing the transducer can be easily defined. However, this method requires weeks of training in advance, which limits its use in real treatment as well.

In this work, we present an optimization algorithm that can automatically find the optimal position for the single-element transducer to minimize defocusing and focus shift. The optimization algorithm utilizes a full-wave simulation tool called k-Wave to simulate the ultrasound field through the skull. The position of the transducer is adjusted based on the simulation results until a position with minimum defocusing and acceptable focus shift is found. A single-element focused transducer is also guided by a neuro-navigation system (Zeta, Zeta Surgical Inc., Boston, MA, USA) to the planned location, tracking the error in real-time [21].

## 2. Materials and Methods

The algorithm we present finds the optimal location in 3D by comparing simulation results of placing the transducer at different locations. Specifically, the ultrasound fields within the skull cavity with the transducer placed at different locations are simulated. Then, the obtained ultrasound fields are analyzed to quantify the defocusing and focus shift. Between focus shift and defocusing, the former is much more concerning in treatment because it can lead to off-target tissue disruption. Therefore, our optimization works by first filtering out locations whose focus shift is greater than a chosen threshold and then finding the location with the smallest defocusing within this subset.

### 2.1. Transcranial Ultrasound Simulation in k-Wave

The acoustic simulations we use to find the optimal transducer location employ a tool called k-Wave, which is a MATLAB toolbox designed for the time-domain simulation of propagating acoustic waves [22]. It has been widely used and studied in ultrasound field and is well accepted as the most accurate simulation tool for transcranial ultrasound applications [23].

There are four input structures in k-Wave, including ‘kgrid’, ‘medium’, ‘source’, and ‘sensor’. These inputs create the computational grid, the material properties of the medium for acoustic waves to propagate, the properties and location of the acoustic source in the medium, and the properties and locations of the sensor used to record the acoustic field, respectively. Among the four input structures, ‘kgrid’, ‘source’, and ‘sensor’ can be defined by using built-in functions and known transducer specifications, while the ‘medium’ inputs require a digital model of the skull. This can be derived from a 3D medical scan of the cranium using the methods described in the following section.

#### 2.1.1. Define ‘Medium’ in k-Wave

To define ‘medium’ for transcranial ultrasound simulations, CT images are normally used to give information of the internal structure of the skull bone. Specifically, the information of density, speed of sound, absorption, and geometry of the skull are abstracted from CT images and added into k-Wave for simulation [13,24,25]. Hounsfield Units (HU) are used to derive a map of porosity of the skull. The HU is defined as follows:

(1) $$HU=1000\times \frac{\mu_{x}-\mu_{water}}{\mu_{bone}-\mu_{water}}$$

where $\mu_{x}$, $\mu_{water}$, and $\mu_{bone}$ are the photoelectric linear attenuation coefficients in tissue, water, and bone, respectively. Then, a linear change in porosity (ϕ) is assumed corresponding to the change in the attenuation coefficients of tissue, water, and bone. Hence, the attenuation coefficient of tissue can be represented as follows:

(2) $$\mu_{x}=\phi \mu_{water}+\left(1-\phi \right)\mu_{bone}$$

In this case, we can directly link the porosity map to HU:

(3) $$\phi =1-\frac{HU}{1000}$$

Then, the other acoustic properties of the skull can be deduced from the density map:

(4) $$\begin{array}{cc} \rho & =\phi \rho_{min}+\left(1-\phi \right)\rho_{max}\\ c & =c_{min}+\left(c_{max}-c_{min}\right)\left(1-\phi \right)\\ \alpha & =\alpha_{min}+\left(\alpha_{max}-\alpha_{min}\right)\left(1-\phi \right) \end{array}$$

where $\rho_{min}$, $c_{min}$, and $\alpha_{min}$ are, respectively, the density, speed of sound, and reference absorption value of water; and $\rho_{max}$, $c_{max}$, and $\alpha_{max}$ are, respectively, the density, sound speed, and reference absorption value of cortical bone. With this information, each voxel in the 3D simulation is allocated with its acoustic properties. The values of the acoustic properties are as follows: $\rho_{min}$ = 1000 kg/m^3^, $\rho_{max}$ = 2200 kg/m^3^, $c_{min}$ = 1500 m/s, $c_{max}$ = 2800 m/s, $\alpha_{min}$ = 0 Np/m, and $\alpha_{max}$ = 20 Np/m [26]. The CT data we used in the simulation had a resolution of 0.5 in all three dimensions. The pixel size of the CT was adjusted using interpolation to be the same as the voxel size of the simulation.

#### 2.1.2. Specifications of the Single-Element Focused Transducer

The specifications of the single-element focused transducer defined in k-Wave are as follows. The transducer has an aperture size and radius of curvature of 10 cm. We used the built-in function called ‘makeBowl’ to create the single-element focused transducer in k-Wave. The frequency of the transducer is 0.5 MHz. The input signal is 10 cycles of toneburst source.

#### 2.1.3. Accuracy of k-Wave Simulation

Before accurately simulating the ultrasound field, convergence tests need to be run on k-Wave for finding the optimal parameters to minimize systematic error. The most important factor that affects the accuracy of the k-Wave results is point per wavelength (PPW). In convergence tests, the resulting pressure, deviation, and focal volume are monitored while the PPW is increased. The sufficient PPW is found until the pressure, deviation, and focal volume are stable.

In this study, we selected 5 PPW for all the simulations. This PPW was chosen for simulating with a good speed and with a reasonably good accuracy. Based on a comprehensive study on k-Wave simulation, the ultrasound parameters we used (0.5 MHz and toneburst 10 cycles) with 5 PPW had an acceptable accuracy [27]. Specifically, the accuracy in pressure was approximately 90%, the deviation was 0.2 mm, and the error in focal volume was approximately 10%. The resolution of the skull property map was adjusted accordingly using the method described above.

To further reduce the error caused by placing the transducer at different locations, we ran simulations both in water and through a skull for every location. The differences between the results of in-water and through-skull simulations were analyzed and used for finding the optimal transducer location.

### 2.2. Optimization Algorithm

The purpose of this optimization algorithm is to find the optimal location to place the transducer so that the transducer can focus ultrasound at the intended target with an acceptable focus shift and minimal defocusing.

**Figure 1:**
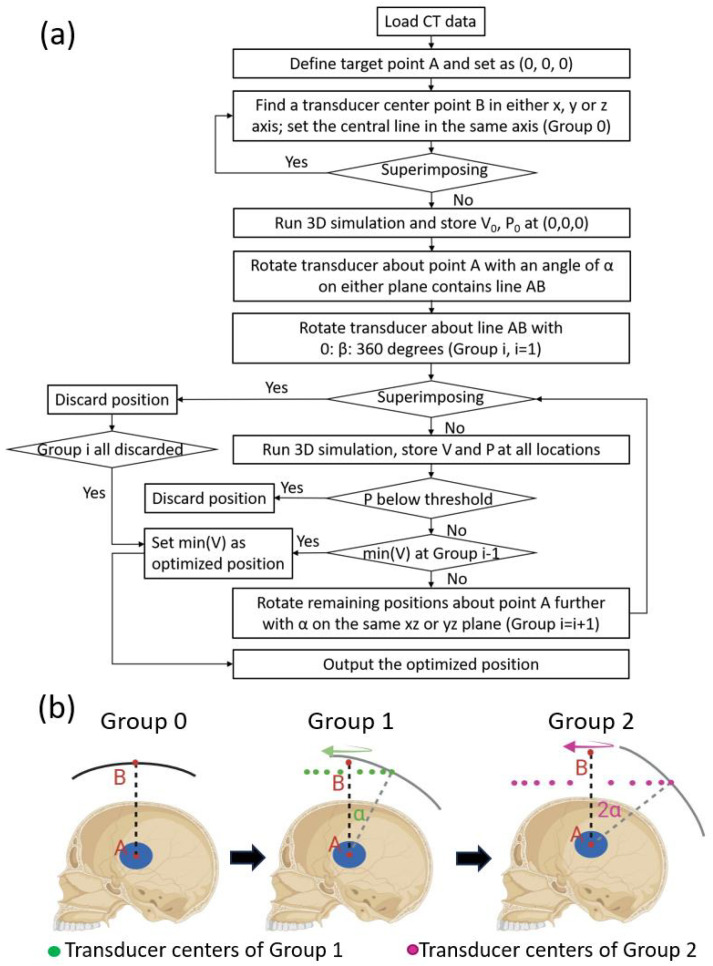
Workflow of the optimization algorithm. (**a**) CT data of the patient are first loaded, and the center point of the target is defined as the origin A (0,0,0). Then, the algorithm finds the center point of the transducer B in the x, y, or z axis. This point B is used to define the first transducer location, which is defined as Group 0. A simulation is run for the Group 0 location, and the resulting PNP and FWHM focal volume are defined as P0 and V0, separately. Then, the transducer center is rotated around the origin A on either the sagittal, transverse, or coronal plane by a degree of α, which creates a new transducer center. The new transducer center is then rotated about line AB for 360° in a degree step of β, creating several locations defined as Group 1. The locations in Group 1 are simulated, then the results (P1 and V1) are analyzed to find a location with an acceptable focal shift and the least defocusing, which is compared to Group 0 to find the optimal location. If the optimal location is in Group 0, then the algorithm stops; if not, then the algorithm continues to create Group 2, following the same steps for creating Group 1. The locations of Group 2 are then simulated and compared to Group 1. If the optimal location is in the former group, then the algorithm stops and outputs the optimal location; if not, the algorithm continues to Group 3 until it finsd the optimal location or runs out of new locations. (**b**) An example of the process of creating different location groups.

The scheme of the algorithm is shown in Figure 1. CT data of the skull are first uploaded, processed, and used to define the medium in the simulation. Then, the center of the sonication region is defined as point A and set to (0,0,0). All the locations simulated were divided into several groups.

Group 0: the algorithm first finds a transducer center point B in either the x, y, or z axis. Then, the transducer is defined in the simulation and superimposing between the transducer and the skull is checked. If there is any superimposition, the algorithm goes back to the former step to find another transducer center point B. Otherwise, the 3D simulation is run and the resulting ultrasound field is analyzed. The focal volume and peak-negative pressure (PNP) of the starting position are stored as $V_{0}$ and $P_{0}$, respectively. PNP is used, as it is directly associated with therapeutic effects like caviation and tissue interaction. Point B is the center point of the first transducer location, not the center point of any other transducer locations after being rotated from the first location.

Group 1: after Group 0 is simulated, the transducer is first rotated about point A with an angle of α (on either the xy, yz, or xz plane that contains line AB), which is 20° in this study. The transducer is then rotated about line AB for 360° at a step size of β. The step size is 20° in this study, leading to 18 transducer locations for this group. Three-dimensional simulations are run for all these locations after discarding the locations with superimposition. After processing and storing the PNP and focal volume for all locations, the focus shift is first analyzed. A threshold on the normalized PNP at the focal point was set beforehand, which was 0.95 in this study. A normalized PNP smaller than 0.95 is considered a large focus shift; hence, such locations are discarded. The smallest full width at half maximum (FWHM) focal volume of the remaining locations is then identified, which represents the optimal location with the best focal quality.

Group 2: if the optimal location is in Group 0 instead of Group 1, the algorithm will terminate after Group 1 and output the optimal location. Otherwise, the algorithm continues by rotating the remaining locations in Group 1 with another angle of α to form Group 2 locations. The Group 2 locations are simulated and compared to Group 1. If the location with an acceptable focus shift and minimal defocusing appears in Group 2 (the new group), then the algorithm continues to Group 3. Otherwise, the best location in Group 1 will be output as the optimal location.

The algorithm terminates when the optimal location is found in the second-last group, or when the locations within the last group are all discarded where the best location in the second-last group is also output as the optimal location.

### 2.3. Robotic Navigation System

**Figure 2:**
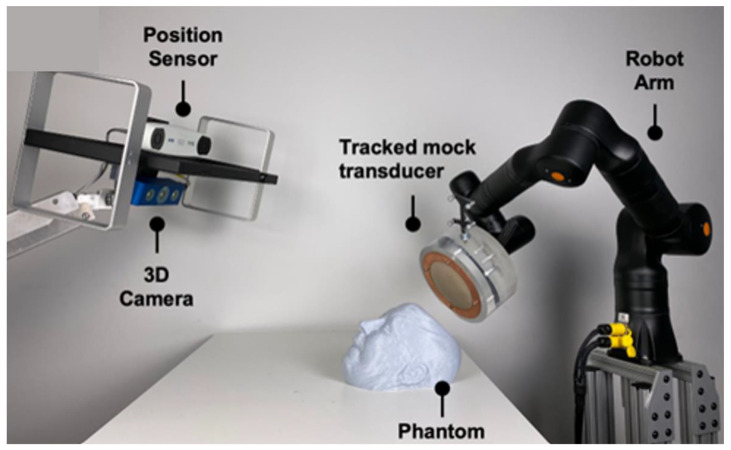
Robotic-guided system. Navigation and robotic setup using the Zeta navigation system and a robotic arm to map the phantom’s face and automatically place the model transducer.

The Zeta Cranial Navigation System (Zeta Surgical Inc., Boston, MA, USA) was used to guide and automate the placement of the transducer at the optimal location. The system uses a 3D camera to capture the phantom’s facial features, which it then uses to register their CT or MRI images in real-time to enable motion-aware navigation using its Real-Track feature. The system was further combined with the KR810 robotic arm (Kassow Robots, Kajakvej, Denmark), allowing the platform to track a phantom head using the robotic arm in real-time (Figure 2).

## 3. Results

### 3.1. k-Wave Simulation

#### 3.1.1. Simulation of Group 0

**Figure 3:**
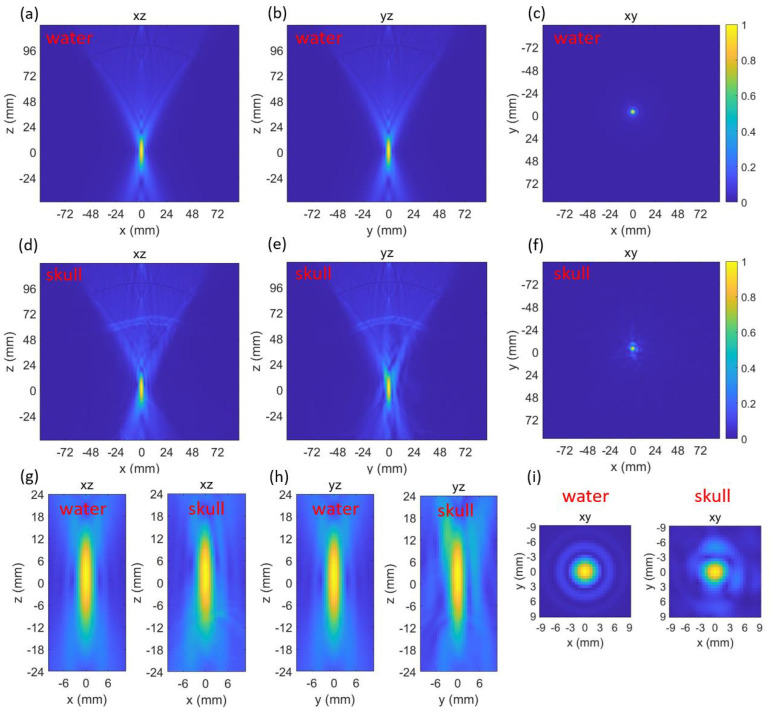
Three-dimensional k-Wave results at the first location while in water (**a**–**c**) and through skull (**d**–**f**). Zoomed-in comparisons of the focus in water and through skull (**g**–**i**).

Both the beam profiles generated by the transducer in water and through skull were simulated and plotted (Figure 3). The normalized peak-negative pressure (PNP) at the targeted point (0,0,0) in water and through the skull were 0.992 and 0.988, respectively. The full width at half maximum (FWHM) focal volume of the beam profile in water and through the skull were 282.7 mm^3^ and 344.3 mm^3^, respectively. The insertion of the skull caused negligible effects on the normalized PNP, which means the focus shift could be ignored. However, the presence of the skull increased the focal volume by 21.8%.

#### 3.1.2. Simulation of Group 1

The k-Wave results of the Group 1 locations in water and through the skull are listed in Table 1. There were 18 locations in Group 1. The average of the normalized PNP and FWHM focal volume in water at the targeted point were 0.998 ± 0.0021 (SD) and 286.73 ± 2.37 (SD) mm^3^, respectively. The small variation in the normalized PNP indicated negligible error caused by different locations, while the FWHM focal volume in water had a slightly larger difference due to placing the transducer at different locations. Both the normalized PNP and FWHM focal volume in water were symmetric about 90°, 180°, 270°, and 360°.

**Figure 4:**
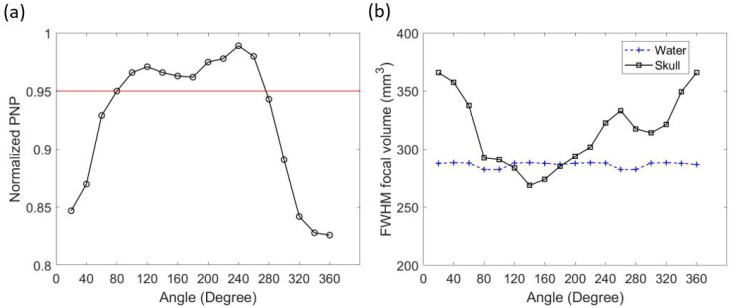
k-Wave results of transducer locations in Group 1. Normalized PNP through the skull at the focal point and the 0.95 threshold (**a**). FWHM focal volume in water and through the skull (**b**).

**Figure 5:**
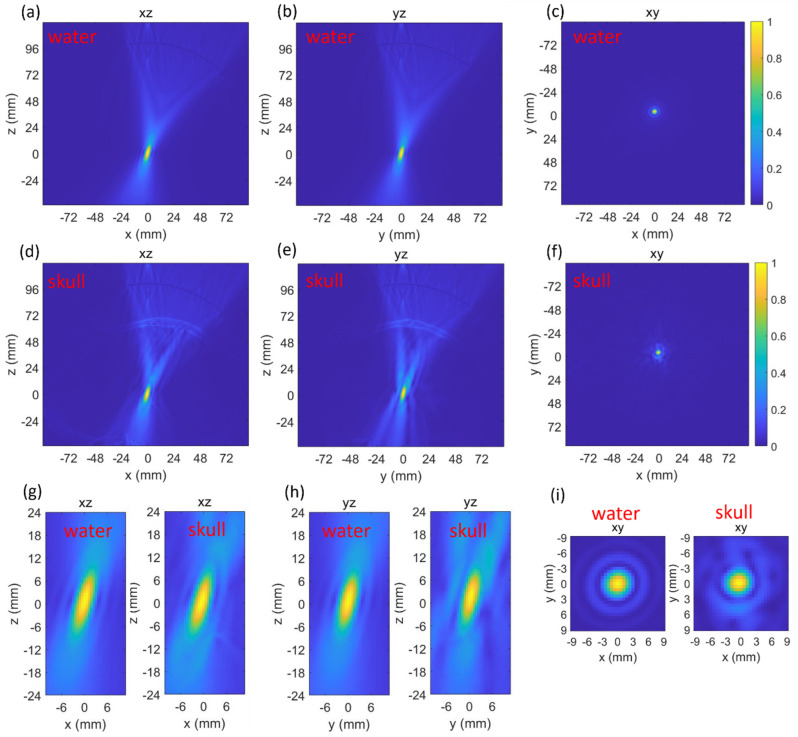
Three-dimensional k-Wave results of the beam profile generated by placing the transducer with an angle of 140° in Group 1. Results in water (**a**–**c**) and through the skull (**d**–**f**). Zoomed-in comparisons of the focus in water and through the skull (**g**–**i**).

While simulating with the skull, the results of different transducer locations were very different. The minimal normalized PNP through the skull at the focal point was 0.826, which was 17.2% smaller than the averaged normalized PNP in water. The normalized PNP at the focal point was above the pre-set threshold (0.95) when the transducer was placed between 80° and 260° in Group 1 (seen in Table 1 and in Figure 4a). The change in FWHM focal volume after inserting the skull heavily depended on the transducer location. The change in focal volume varied from a 27.6% increase to a 6.8% decrease (seen Table 1). When the transducer location was between 120° and 180°, the focal volume through the skull was smaller than in water (Figure 4b). The best focal quality in Group 1 appeared at an angle of 140° (Figure 5). In this location, the normalized PNP was 0.966 (above 0.95), and the FWHM focal volume was the smallest in Group 1 (6.8% smaller than in water).

#### 3.1.3. Simulation of Group 2

The transducer location with the best focal quality in Group 1 (at 140°, with a focal volume 6.8% smaller than in water) is better than Group 0 (21.8% bigger than in water). Therefore, the algorithm continued to take the locations above threshold in Group 1 and generated Group 2. The k-Wave simulation results of the transducer locations in Group 2 are listed in Table 2. There were 10 locations in the group. The normalized PNP at the focal point in water had an average value of 0.999 ± 0.0024 (SD), while the focal volume had an average value of 272.4 ± 2.48 mm^3^. A symmetric pattern in the values was also observed.

**Figure 6:**
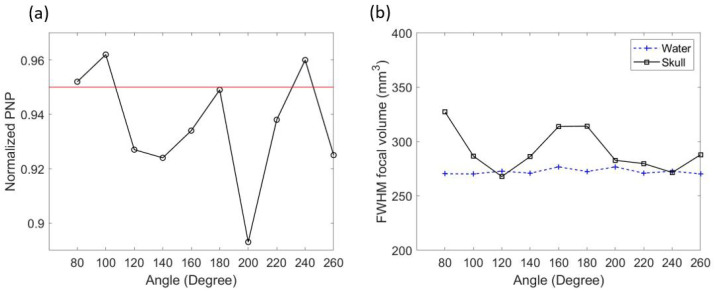
k-Wave results of transducer locations in Group 2. Normalized PNP through the skull at the focal point and the 0.95 threshold (**a**). FWHM focal volume in water and through the skull (**b**).

**Figure 7:**
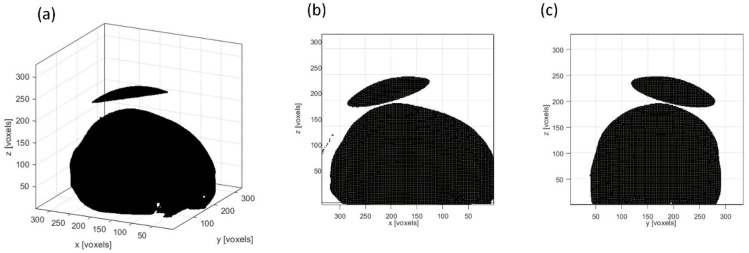
The optimal location for placing the single-element focused transducer. The view in 3D (**a**), in the x–z plane (**b**), and in the y–z plane (**c**).

While through the skull, only three of the ten locations were still above the 0.95 threshold after another rotation (seen in Table 1 and Figure 6a). They were at 80°, 100°, and 240°. The FWHM focal volume showed a larger change after the skull insertion (Figure 6b). The focal volume increased at most locations but also decreased at 120° and 240°. In Group 2, the location with the best focal quality and a PNP above 0.95 was at 240°. However, when comparing to the location with the best focal quality in Group 1 (at 140°, with a focal volume 6.8% smaller than in water), the focal quality in Group 2 was worse. Hence, the transducer location in Group 1 at 140° was the optimal location (Figure 7).

### 3.2. Transducer Placement and Target Tracking via a Surgical Robot

**Figure 8:**
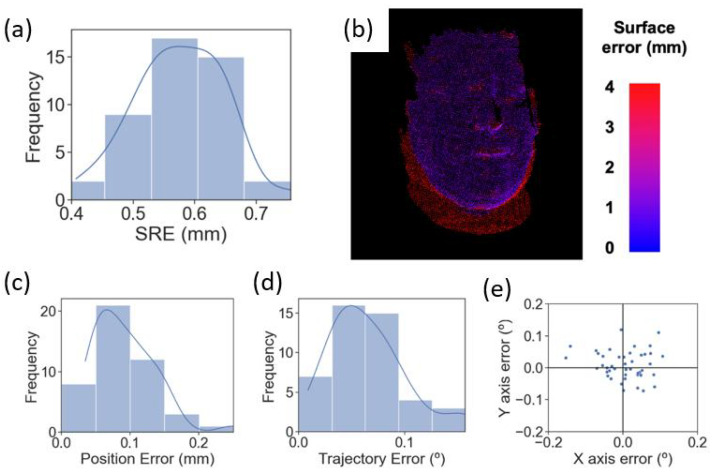
Surface registration error, the average image registration error between each surface point on the phantom’s 3D image and its corresponding point on the CT scan. SRE for 45 independent registrations to the phantom (**a**). Heatmap showing SRE values across the phantom’s face of a representative registration (**b**). PE (**c**) and TE (**d**) distribution for 45 independent robot targets. X and y TE for the 45 targets (**e**).

The Zeta navigation platform was used to guide and automate the placement of the transducer during treatment. Figure 8a,b demonstrates the surface registration error (SRE), which is the deviation in the system’s reconstructed point cloud of the phantom collected by the system’s camera assembly from the uploaded CT scan of an actual human head. Figure 8a shows the SRE for 45 independent registration attempts, resulting in a mean SRE of 0.575 ± 0.0711 mm (SD). Figure 8b shows a heatmap of SRE values across the phantom’s surface of a representative registration attempt. The blue points represent low SRE values between 0 and 1 mm, the red points represent SRE values higher than 3 mm, and the rest are in between. The figure demonstrates that the system can register the phantom face to the CT scan with a low SRE, represented by most blue points observed.

Figure 8c,d demonstrates the surgical robot’s precision in positioning the mock transducer at the optimal location in terms of position error (PE) and trajectory angle (TE). Forty-five independent attempts resulted in a mean PE of 0.0925 ± 0.0431 mm (SD) and a mean TE of 0.0650 ± 0.0352 mm (SD). Figure 8e elaborates on the x-axis error and y-axis error of the TE demonstrated in Figure 8d.

## 4. Discussion

We presented an optimization algorithm for finding the optimal transducer location and used the Zeta navigation platform to automatically place the transducer at the optimal location. The optimization algorithm utilized a MATLAB toolbox called k-Wave to simulate the beam profiles when placing the transducer at different locations. Then, based on the pre-set threshold on normalized PNP and the minimal FWHM focal volume, a transducer location with an acceptable focus shift and the best focal quality was found. The Zeta navigation platform and a robotic arm were used to guide the transducer to the optimal location and track the placement error in real-time.

There are different ways of quantifying the focus shift. The most straightforward and accurate way is to quantify the distance between the targeted point and the actual location with the highest pressure. However, sometimes, this is not necessarily the best way. Both the intended target and the beam profile generated by the transducer are much larger than just one point and are not in a regular shape. The shift of one point is not a good representation of the overlapping status of the intended target and the actual beam profile. Hence, we used a threshold on the normalized PNP here to quantify the focus shift. The higher the normalized PNP at the focal point is, the less the focus is shifted.

The insertion of the skull caused a large increase (21.7%) in the FWHM focal volume (Figure 3) but a negligible decrease in the normalized PNP. This indicated that after penetrating through the skull, the focus shift was negligible, but the focal quality became worse. Such changes indicated that the acoustic pathway at this location was similar in the speed of sound but had a defocusing effect caused by the geometry of the skull. In Group 1, both the changes in focal volume and normalized PNP became much more obvious (Table 1). This was due to the skull geometry and thickness at these locations changing at a large scale. This also indicated the importance of finding the optimal location for a single-element focused transducer. In Groups 1 and 2, there were locations with decreased focal volume. The decrease in focal volume was due to the curvature of the skull acting as a focal lens at those locations.

The threshold on normalized PNP (0.95) and the rotation angles (α = 20°, β = 20°) were selected considering the size of the transducer and the time needed to run the algorithm. A higher threshold on PNP and a smaller rotation angle would lead to a better result. We used the same angles for both Group 1 and Group 2. This could be optimized by using a varying angle, which decreases at good locations and increases at bad locations.

Once the optimal location was determined, the Zeta navigation system and a robotic arm were used to guide and place the mock transducer at the optimal location. The low PE (0.0925 ± 0.0431 mm) and TE (0.0650 ± 0.0352 mm) indicated accurate placement of the transducer during the treatment.

There are limitations in this study. First, instead of running a convergence test, we used the same parameters from the study with detailed test results. We did not run convergence tests because the GPU we used for the simulation did not have a large enough memory. However, we used ultrasound parameters that have been investigated and with an acceptable accuracy with 5 PPW. We also used the comparison between the in-water simulations and through-skull simulations to minimize the error caused by the low PPW. With a high enough PPW, this algorithm can be run without the need for running in-water simulations. Second, the rotation angles we used could be smaller to obtain a better result. However, as an indication of the algorithm, we successfully found the optimal location to place the transducer. The algorithm can also be optimized for both being more efficient in time and being able to search using a finer step size. Third, the shape of the beam profile (focus) generated by the transducer was not considered. The focus generated by a single-element focused transducer is normally in a long oval shape. Hence, the angle of the transducer affects the actual treated region. Therefore, this algorithm can be optimized by taking the size and shape of the diseased tissues into consideration. The presented algorithm is different from exhaustive search. Although this algorithm requires a number of simulations, it can stop before running all possible locations due to the grouping and comparison within different groups, which saves time.

## 5. Conclusions

We presented an optimization algorithm that is capable of finding the optimal location for placing a single-element focused ultrasound for transcranial ultrasound treatment. The algorithm simulates the beam profiles generated by the transducer at different locations, then compare the results to find the location with an acceptable focus shift and minimal defocusing. This location is output as the optimal location for placing the transducer. A neuro-navigation system (Zeta) and a robotic arm are used to guide and automatically place the transducer beside the patient (head phantom) with good accuracy. This algorithm and placement setup can make transcranial focused ultrasound treatment safer and more successful.

## Figures and Tables

**Table 1:** Simulation results of Group 1 locations in water and through the skull.

Angle (°)	Normalized PNP		FWHM Focal Volume mm^3^		
	Water	Skull	Water	Skull	Change
20	0.999	0.847	287.9	365.9	27.1%
40	0.999	0.870	288.4	357.7	24.0%
60	0.999	0.929	288.1	337.6	17.2%
80	0.994	0.950	282.5	292.5	3.5%
100	0.994	0.966	282.5	291.2	3.1%
120	0.999	0.971	288.1	283.8	−1.5%
140	0.999	0.966	288.4	268.9	−6.8%
160	0.999	0.963	287.9	274.1	−4.8%
180	0.999	0.962	286.8	285.3	−0.5%
200	0.999	0.975	287.9	293.8	2.0%
220	0.999	0.978	288.4	301.6	4.6%
240	0.999	0.989	288.1	322.5	11.9%
260	0.999	0.980	282.5	333.1	17.9%
280	0.994	0.943	282.5	317.5	12.4%
300	0.994	0.891	288.1	313.9	9.0%
320	0.999	0.842	288.4	321.2	11.4%
340	0.999	0.828	287.9	349.5	21.4%
360	0.999	0.826	286.8	365.9	27.6%

**Table 2:** Simulation results of Group 2 locations in water and through the skull.

Angle (°)	Normalized PNP		FWHM Focal Volume mm^3^		
	Water	Skull	Water	Skull	Change
80	0.995	0.952	270.4	327.2	17.4%
100	0.995	0.962	270.2	286.6	5.7%
120	1	0.927	272.8	267.8	−1.9%
140	1	0.924	270.9	286.2	5.3%
160	1	0.934	276.7	313.8	11.8%
180	1	0.949	272.4	314.1	13.3%
200	1	0.893	276.7	282.7	2.1%
220	1	0.938	270.9	279.7	3.1%
240	1	0.960	272.8	271.5	−0.5%
260	0.995	0.925	270.2	287.9	6.1%

## Data Availability

The data presented in this study are available on request from the corresponding author. The data are not publicly available due to privacy reasons.
